# Injection drug use and sexually transmitted infections among men who have sex with men: A retrospective cohort study at an HIV/AIDS referral hospital in Tokyo, 2013–2022

**DOI:** 10.1017/S0950268823001772

**Published:** 2023-11-15

**Authors:** Kazuhiko Ikeuchi, Makoto Saito, Eisuke Adachi, Michiko Koga, Kazuya Okushin, Takeya Tsutsumi, Hiroshi Yotsuyanagi

**Affiliations:** 1 Department of Infectious Diseases and Applied Immunology, IMSUT Hospital of The Institute of Medical Science, The University of Tokyo, 4-6-1 Shirokanedai, Minato-ku, Tokyo, Japan; 2 Division of Infectious Diseases, Advanced Clinical Research Center, Institute of Medical Science, The University of Tokyo, 4-6-1 Shirokanedai, Minato-ku, Tokyo, Japan; 3 Department of Infection Control and Prevention, The University of Tokyo, 7-3-1 Hongo, Bunkyo-ku, Tokyo, Japan

**Keywords:** syphilis (T. pallidum infection), sexually transmitted infections (STIs), injecting drug use (IDU), HIV disease (AIDS), hepatitis C virus (HCV)

## Abstract

Men who have sex with men (MSM) who use injection drugs (MSM-IDU) are at high risk of sexually transmitted infections (STIs), but the long-term incidence is unclear. We conducted a single-centre retrospective cohort study using the clinical records of non-haemophilia men with human immunodeficiency virus (HIV) who visited the Institute of Medical Science, the University of Tokyo (IMSUT) Hospital, located in Tokyo, Japan, from 2013 to 2022. We analysed 575 patients including 62 heterosexual males and 513 MSM patients, of whom 6.8% (35/513) were injection drug use (IDU). Compared to non-IDU MSM, MSM-IDU had a higher incidence of hepatitis C virus (HCV) (44.8 vs 3.5 /1,000 person-years (PY); incidence rate ratio (IRR) [95% confidence interval (95% CI)], 12.8 [5.5–29.3], *p* < 0.001) and syphilis (113.8 vs 53.3 /1,000 PY; IRR, 2.1 [1.4–3.1], *p* < 0.001). The incidence of other symptomatic STIs (amoebiasis, chlamydia, and gonorrhoea infections) was <4/1,000 PY. In multivariable Poisson regression analysis, HCV incidence was associated with MSM (IRR, 1.8 × 10^6^ [9.9 × 10^5^–3.4 × 10^6^], *p* < 0.001), IDU (IRR, 10.1 [4.0–25.6], *p* < 0.001), and syphilis infection during the study period (IRR, 25.0 [1.2–518.3]/time/year, *p* < 0.001). Among men with HIV, the prevalence of IDU in MSM and the long-term incidence of STIs in MSM-IDU were high. IDU and sexual contact are important modes of transmission of HCV among HIV-infected MSM in Tokyo.

## Introduction

There are two major human immunodeficiency virus (HIV) transmission routes in developed countries: injection drug use (IDU) and sexual contact, particularly among men who have sex with men (MSM) [1]. MSM with HIV are at higher risks of sexually transmitted infections (STIs), such as syphilis, amoebiasis, and hepatitis A virus (HAV) [2–4], and injection drug users with HIV are at a higher risk of bloodborne infections, such as hepatitis B virus (HBV) and hepatitis C virus (HCV) [5, 6]. HBV and HCV are also transmitted by sexual contact, especially among MSM [6, 7]. However, some MSM use illicit drugs recreationally during sex, called ‘chemsex’. Crystalline methamphetamine, gamma-hydroxybutyric acid, gamma-butyrolactone, and mephedrone are commonly used for chemsex [8, 9]. Among them, the practice of injecting drugs during sexual contact is termed ‘slamsex’. MSM are more likely to engage in chemsex and slamsex, have multiple partners, have unprotected sex, and share needles [8–10]. MSM who use injection drugs (MSM-IDU) are reported to have a higher incidence of HIV and a higher prevalence of non-bloodborne STIs, such as syphilis and gonorrhoea [8–13]. However, the long-term incidence of repeated STIs in MSM-IDU has not yet been thoroughly investigated.

It is of particular importance to reveal the transmission route of HCV among the various STIs. The transmission rate of HCV is estimated at 1/190,000 heterosexual sexual contacts [14]; therefore, HCV is considered to be transmitted mainly by injection drugs in developed countries. However, recent outbreaks of HCV among HIV-infected MSM have been reported worldwide, indicating that HCV can be more easily transmitted through male-to-male sexual contacts [15–18]. Although direct-acting antivirals (DAAs) can achieve sustained virological response (SVR) against HCV in >90% [19], the high reinfection rate in people living with HIV (PLWH) is of great concern [20]. Understanding the HCV transmission route is essential for elimination efforts.

Japan has one of the lowest IDU prevalence rates among the developed countries. Unlike in the United States, heroin users are extremely rare in Japan, and almost all illegal drugs used for injection are methamphetamine, both in the general population and among MSM with HIV [21–23]. Most arrests for illicit injection drugs involve methamphetamine, which is punishable in Japan by imprisonment [22]. The lifetime experience rate of methamphetamine is only 0.5% among the general population in Japan and 2–10% in the United States and European countries [23]. However, the amount of methamphetamine imported into Japan has more than doubled over the past five years [23]. The recent prevalence of IDU among MSM and their sexual behaviour is not well understood in Japan.

We conducted a single-centre retrospective cohort study of PLWH describing the prevalence of IDU patients and the seroprevalence and long-term incidence of STIs among MSM patients with HIV in Tokyo, Japan. Our secondary objective was to investigate the factors associated with major STIs and compare them between MSM-IDU and non-IDU MSM.

## Methods

### Setting and participants

We retrospectively reviewed routinely collected clinical records of PLWH who had at least one outpatient visit from January 2013 to April 2022 at the Institute of Medical Science, the University of Tokyo (IMSUT) Hospital, an HIV/acquired immune deficiency syndrome (AIDS) referral hospital in Tokyo, Japan. We included PLWH who underwent both HCV antibody (Ab) and *Treponema pallidum* Ab (TPAb) tests in the study period. We excluded female patients, patients with haemophilia, and patients whose sexual behaviour was unknown. The participants were divided into three groups: (1) heterosexual males, (2) non-IDU MSM, and (3) MSM-IDU. The baseline characteristics assessed at the first visit during the study period and the incidence of STIs were compared between the three groups.

This study was approved by the Institutional Review Board of the Institution of Medical Science, University of Tokyo (approval number: 2022-48-1128), and was conducted using an opt-out method to obtain informed consent from the hospital website (https://www.ims.u-tokyo.ac.jp/imsut/content/000007228.pdf).

### Data extraction and definition of outcomes

We extracted the following variables assessed at baseline: age, race, sexual behaviour, IDU, observation period, time from antiretroviral treatment (ART) initiation, time from the first visit to the IMSUT hospital, cluster of differentiation 4 (CD4) count, HIV viral load (<50 copies/mL), antiretroviral therapy use, TPAb, HCV Ab, and hepatitis B core Ab (HBcAb). Seropositivity for syphilis, HCV, and HBV was determined based on positive results for TPAb, HCV Ab, and HBcAb, respectively.

In this study, an injection drug user was defined as any patient who had ever used an injection drug as confirmed by a medical interview or by any arrest record for illegal drug use. During their first visit to the hospital, all PLWH underwent an interview with a physician to ascertain whether they had a history of IDU. However, owing to the strong stigma and illegality of methamphetamine use in Japan, patients often do not disclose their current drug use. In other situations, IDUs are often discovered when patients are arrested for injecting drug use or treated for drug addiction. In Japan, when a patient with HIV is arrested, the police usually contact physicians to prescribe antiretrovirals. Even when the police do not contact the hospital, outpatient visits are interrupted; consequently, almost all arrests (for illegal drug use) are practically detectable. Information on IDU patients who were neither disclosed nor arrested was not captured in this study, which could have underestimated the prevalence of IDU.

The incidence of syphilis, hepatitis C, amoebiasis, hepatitis A, chlamydia, and gonorrhoea was evaluated. The diagnosis of STIs was ascertained by two infectious disease specialists. Syphilis reinfection was defined as a new infection after a >fourfold decrease in the rapid plasma reagin titre following treatment. HCV reinfection was defined as HCV ribonucleic acid (RNA) becoming positive any time after 12 weeks since SVR was achieved.

At our hospital, outpatient laboratory data and clinical histories were shared among HIV doctors at weekly meetings to ensure that Ab testing for syphilis, HAV, HBV, and HCV was conducted annually, particularly for sexually active patients, although the physician who observed the patient made the final decision to conduct the tests. Testing for chlamydia, gonorrhoea, and amoebiasis was performed only when the patients exhibited relevant symptoms. Nucleic acid amplification testing of the urine was performed for chlamydia and gonorrhoea.

### Statistical analysis

The median with interquartile range (IQR) or proportion (%) was used to describe continuous or categorical variables, respectively. Using the Mann–Whitney U test or Fisher’s exact test, baseline characteristics were first compared between heterosexual males and MSM and then between MSM-IDU and non-IDU MSM. The incidence rate ratio (IRR) with a 95% confidence interval (95% CI) between the groups was calculated using the mid-P exact method. The observation period in person-years (PY) at risk was defined as the period from the first visit to the last visit during the study period, accounting for the risk of repeated infection except for HAV and HCV. As reinfection does not occur in HAV, the PY at risk for calculating HAV incidence was defined from the first visit to the last visit or until HAV infection. To calculate the incidence of HCV reinfection, the time from the first HCV infection to 12 weeks after SVR was excluded from the PY at risk. STIs diagnosed concurrently with HIV infection at the first visit were excluded from the calculation of the incidence.

The characteristics associated with HCV infection and syphilis were also explored. The incidence rates of newly diagnosed HCV and syphilis during the observation period were analysed using Poisson regression or negative binomial regression, as appropriate. The variables for the multivariable model were selected by backward elimination using the Wald test (*p* < 0.05) with a robust variance estimator. MSM and IDU were included regardless of statistical significance, based on a priori interest. A complete case analysis was performed. Data analyses were performed using R version 4.0.2. or Stata MP 16.1 (StataCorp, Texas, USA).

## Results

**Figure 1.**
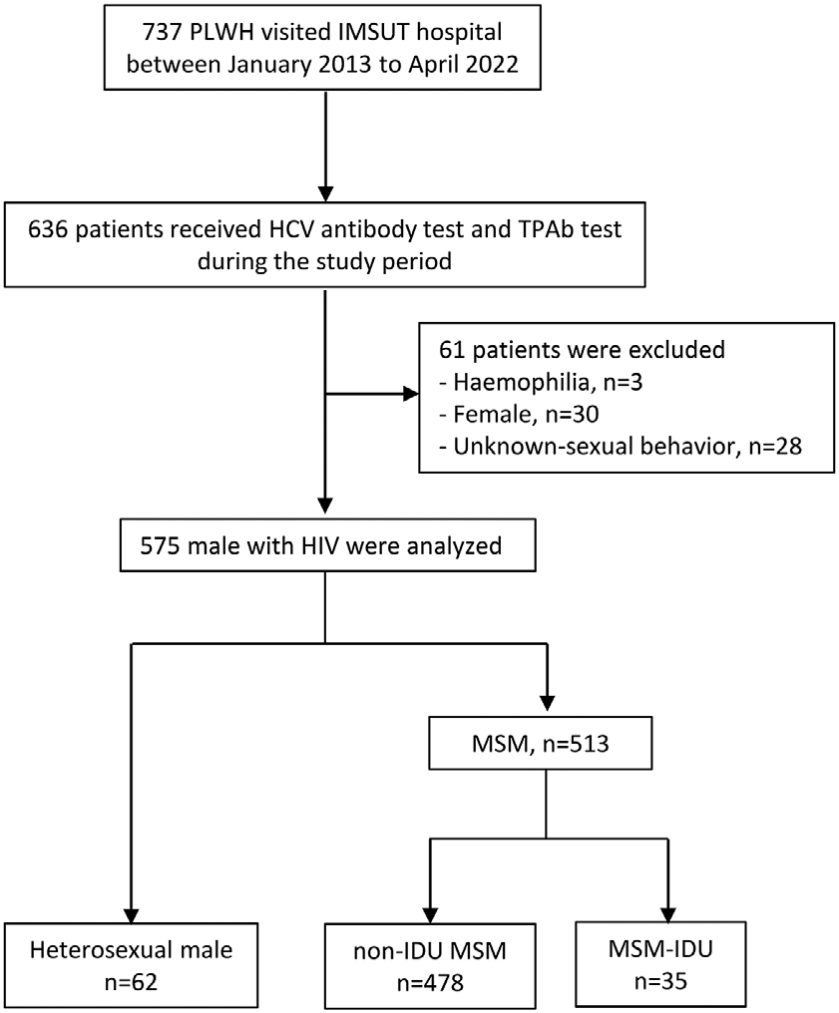
Study flow chart. Abbreviations: HCV, hepatitis C virus; IDU, injection drug use; MSM, men who have sex with men; PLWH, people living with HIV.

From January 2013 to April 2022, 737 PLWH visited IMSUT Hospital (Figure 1), among whom, 636 patients underwent both HCV Ab and TPAb tests, and 61 patients were excluded (haemophilia, n = 3; female, n = 30; unknown sexual behaviour, n = 28). A total of 575 patients, including 62 heterosexual males, 478 non-IDU MSM patients, and 35 MSM-IDU patients, were analysed. The observational period was almost the same between the three groups (heterosexual group, 8.9 [4.8–9.1] years; non-IDU MSM, 8.9 [5.4–9.1] years; and MSM-IDU, 8.9 [6.7–9.1] years). At the end of the study period, 97% (560/575) of the patients were treated with ART, and 89.9% (517/575) were well-controlled (HIV RNA < 50 copies/mL).

Patient characteristics at the initial visit during the observation period are shown in Table 1. Compared with MSM, heterosexual male patients were older (heterosexual male vs MSM, median 45 [IQR 37–56] vs 40 [34– 47] years, *p* = 0.002), less likely to be Asian (88.7% [55/62] vs 95.7% [491/513], *p* = 0.03), and had lower CD4 counts (354 [237–509] vs 434 [302–587], *p* = 0.01). IDUs were less frequent in the heterosexual group, although the difference was not statistically significant (1.6% [1/62] vs 6.8% [35/513], *p* = 0.16). The prevalence of HBcAb (41.9% [26/62] vs 57.1% [293/513], *p* = 0.03) and TPAb (27.4% [17/62] vs 54.8% [281/513], *p* < 0.001) was significantly lower in heterosexual males.

**Table 1.** Patient characteristics at the first visit during the study period

Baseline characteristic	Heterosexual male	MSM				
		All MSM		Non-IDU	IDU	
	n = 62	n = 513	*p*-valueǂ	n = 478	n = 35	*p*-value‡
Age (year)	45 (37–56)	40 (34–47)	0.002	40 (34–47)	42 (35–46)	0.88
IDU	1.6% (1/62)	6.8% (35/513)	0.16	–	–	–
Asian	88.7% (55/62)	95.7% (491/513)	0.03	95.4% (456/478)	100% (35/35)	0.39
Time from ART initiation (year)*	2.4 (0–9.7)	2.6 (0–6.4)	0.65	2.5 (0–6.4)	3.4 (1.3–6.8)	0.15
Time from first visit (year)	5.2 (0.2–11.7)	3.8 (0–8.2)	0.17	3.3 (0–8.1)	5.5 (2.7–8.8)	0.06
CD4 –/μL	364 (237–509)	434 (302–587)	0.01	430 (296–580)	495 (382–599)	0.06
HIV RNA <50 copies/mL	61.3% (38/62)	65.9% (338/513)	0.48	65.9% (315/478)	65.7% (23/35)	1.00
Antiretroviral treatment	69.4% (43/62)	70.6% (362/513)	0.88	69.9% (334/478)	80.0% (28/35)	0.25
HCV Ab	0% (0/62)	3.1% (16/513)	0.24	2.5% (12/478)	11.4% (4/35)	0.02
HBcAb	41.9% (26/62)	57.1% (293/513)	0.03	56.5% (270/478)	65.7% (23/35)	0.38
TPAb	27.4% (17/62)	54.8% (281/513)	<0.0001	51.9% (248/478)	94.3% (33/35)	<0.0001

*Note:* Percentage (number) or median (interquartile range) are shown. *only those who received ART are included. ǂ*p*-values comparing the heterosexual group and the whole MSM group. ‡*p*-values comparing the MSM-IDU group and the non-IDU MSM group.

Abbreviations: Ab, antibody; ART, antiretroviral therapy; HBc, hepatitis B core; HCV, hepatitis C virus; IDU, injection drug use; MSM, men who have sex with men; TPAb, *Treponema pallidum* antibody.

Among the 513 MSM, we compared the characteristics of 35 MSM-IDUs and 478 non-IDU MSM. Baseline characteristics were almost similar between the two groups except for the prevalence of HCV Ab positivity (11.4% [4/35] vs 2.5% [12/478], *p* = 0.02) and TPAb positivity (94.3% [33/35] vs 51.9% [248/478], *p* < 0.001), which were significantly higher in the MSM-IDU group.

The number of new STI cases during the study period is presented in Table 2. Twenty-one patients experienced 23 HCV infections: no heterosexual male (0/62, 0%), 12 non-IDU MSM (12/478, 2.5%), and nine MSM-IDU (9/35, 25.7%). Among them, one MSM-IDU patient had a history of prior HCV infection and was successfully treated with DAA before the observation period. By the end of the study period, 35 patients (35/578, 6.1%) were HCV-seropositive, including no heterosexual male (0/62, 0%), 24 non-IDU MSM (24/478, 5.0%), and 11 MSM-IDUs (11/35, 31.4%). Seven patients with HCV were diagnosed concurrently with HIV, 28 were diagnosed with HCV after HIV infection, and no patients were diagnosed with HCV before HIV infection. Excluding the three patients who cleared HCV spontaneously, 55.5% (10/18) had genotype 1 and 33.3% (6/18) had genotype 2, which was similar to values previously reported for PLWH in Japan [24]. Sixteen patients were treated with DAA, and 15 (93.8%, 15/16) achieved SVR. Among them, two MSM-IDUs were reinfected with HCV during the observational period.

**Table 2.** Incidence rate of sexually transmitted infections

		MSM						
	Heterosexual male	All MSM			Non-IDU MSM	MSM-IDU		
	n = 62	n = 513	IRR (95% CI)	*p*-value	n = 478	n = 35	IRR (95% CI)	*p*-valueǂ
HCV								
Patient number (%)	0 (0%)	21 (4.1%)			12 (2.5%)	9 (25.7%)		
Infection number	0	23			12	11		
PY at risk-year	425.3	3658.7			3413.1	245.6		
Incidence rate –/1,000 PY	0	6.3	Infinite (0.8–infinite)	0.08	3.5	44.8	12.8 (5.5–29.3)	<0.0001
Syphilis								
Patient number (%)	7 (11.3%)	135 (26.3%)			117 (24.5%)	18 (51.4%)		
Infection number	13	213			183	30		
PY at risk-year	425.3	3696.4			3432.8	263.6		
Incidence rate –/1,000 PY	30.6	57.6	1.9 (1.1–3.4)	0.02	53.3	113.8	2.1 (1.4–3.1)	0.0004
Amoebiasis								
Patient number (%)	1 (1.6%)	11 (2.1%)			9 (1.9%)	2 (5.7%)		
Infection number	1	14			11	3		
PY at risk-year	425.3	3696.4			3432.8	263.6		
Incidence rate –/1,000 PY	2.4	3.8	1.4 (0.29–34.5)	0.73	3.2	11.4	3.7 (0.8–12.0)	0.09
HAV								
Patient number (%)	1 (1.6%)	13 (2.5%)			12 (2.5%)	1 (2.9%)		
Infection number	1	13			12	1		
PY at risk-year	421.8	3655.9			3396.1	259.8		
Incidence rate –/1,000 PY	2.4	3.6	1.3 (0.3–32.3)	0.78	3.5	3.8	1.2 (0.1–6.3)	0.85
Chlamydia								
Patient number (%)	0 (0%)	11 (2.1%)			11 (2.3%)	0 (0%)		
Infection number	0	11			11	0		
PY at risk-year	425.3	3696.4			3432.8	263.6		
Incidence rate –/1,000 PY	0	3.0	Infinite (0.4–infinite)	0.30	3.2	0	0 (0–4.1)	0.44
Gonorrhoea								
Patient number (%)	0	3 (0.6%)			3 (0.6%)	0 (0%)		
Infection number	0	3			3	0		
PY at risk-year	425.3	3696.4			3432.8	263.6		
Incidence rate –/1,000 PY	0	0.8	Infinite (0.07–infinite)	0.72	0.9	0	0 (0–22.3)	0.80

*Note:* ǂ*p*-values comparing the heterosexual group and the whole MSM group. ‡*p*-values comparing the MSM-IDU group and the non-IDU MSM group.

The total risk period was 4,083.9 PY for HCV, 4,077.8 PY for HAV, and 4,121.6 PY for other STIs.

Abbreviations: CI, confidence interval; HAV, hepatitis A virus; HCV, hepatitis C virus; IRR, incidence rate ratio; PY, person-year.

Among the STIs, syphilis was the most common. In total, 142 patients (142/578, 24.6%), including seven heterosexual males (7/62, 11.3%), 117 non-IDU MSM (117/478, 24.5%), and 18 MSM-IDU (18/35, 51.4%), experienced 213 syphilis infections. Among these, 111 (111/142, 78.2%) tested positive for TPAb at baseline. Fifty-five patients had multiple infections during the study period: 33 patients had two infections, 16 patients had three infections, five patients had four infections, and one patient had five infections. By the end of the observation period, 19 (19/142, 13.4%) patients had been infected with syphilis only once in their lives, and the others experienced syphilis reinfection.

The incidence of HCV and syphilis was calculated, and the IRRs were compared between the risk groups (Table 2). The incidence rate of HCV tended to be higher in the MSM group than in the heterosexual male group (6.3 vs 0/1,000 PY; IRR [95% CI], infinity [0.8–infinity], *p* = 0.08) and was significantly higher in the MSM-IDU group than in the non-IDU MSM group (44.8 vs 3.5/1,000 PY; IRR [95% CI], 12.8 [5.5–29.3], *p* < 0.001). The incidence rate of syphilis was significantly higher among the MSM group than the heterosexual male group (57.6 vs 30.6/1,000 PY; IRR [95% CI], 1.9 [1.1–3.4], *p* = 0.02) and was significantly higher in the MSM-IDU group than in the non-IDU MSM group (113.8 vs 53.3/1,000 PY; IRR [95% CI], 2.1 [1.4–3.1], *p* < 0.001). The incidence of amoebiasis was higher in the MSM-IDU group than in the non-IDU MSM group, but the difference was not statistically significant (11.4 vs 3.2/1,000 PY; IRR [95% CI], 3.7 [0.8–12.0], *p* = 0.09). The number of HAV, chlamydia, and gonorrhoea infections was small, and the incidence rates were not significantly different between the groups.

Multivariable analyses were conducted to identify predictive factors for HCV infection (Table 3A) and syphilis (Table 3B). Univariable analysis showed that IDU, MSM, and new syphilis infection during the same observation period were associated with an increased incidence of HCV. In the multivariable Poisson regression, MSM (IRR [95% CI], 1.8 × 10^6^ [9.9 × 10^5^–3.4 × 10^6^], *p* < 0.001), IDU (IRR [95% CI], 10.1 [4.0–25.6], *p* < 0.001), and new syphilis infection (IRR [95% CI], 25.0 [1.2–518.3]/time/year, *p* < 0.001) remained significantly associated with HCV infection.

**Table 3A.** Univariable and multivariable regression analysis for HCV incidence

		Univariable		Multivariable	
Characteristic	N/PY	IRR (95% CI)	*p*-value	IRR (95% CI)	*p*-value
IDU	11/250.6	14.0 (5.94–33.10)	<0.001	10.09 (3.98–25.57)	<0.001
No	12/3833.3	Reference		Reference	
MSM	23/3658.7	5.98 × 10^7^ (3.85 × 10^7^–9.28 × 10^7^)	<0.001	1.84 × 10^6^ (9.87 × 10^5^–3.43 × 10^6^ )	<0.001
No	0/425.3	Reference		Reference	
Asian	22/3934.0	0.84 (0.12–5.99)	0.86		
No	1/149.9	Reference			
Age (years)	23/4083.9	0.97 (0.93–1.01)	0.10		
HIV RNA<50 copies/mL at baseline	15/2959.6	0.71 (0.28–1.82)	0.48		
No	8/1124.3	Reference			
CD4 count (100/μL)	23/4123.2	0.90 (0.74–1.10)	0.31		
On ART at baseline	16/3126.4	0.70 (0.26–1.87)	0.48		
No	7/957.6	Reference			
Time from treatment (year)	23/4083.9	1.02 (0.90–1.14)	0.80		
HCV at baseline	2/97.4	3.90 (0.85–17.82)	0.08		
No	21/3986.5	Reference			
HBV at baseline	18/2357.9	2.64 (0.96–7.25)	0.06		
No	5/1726.0	Reference			
Syphilis new infection (time/year)	23/4083.9	109.5 (7.25–1654.52)	0.001	24.95 (1.20–518.27)	0.04
TPAb at baseline	17/2215.7	2.39 (0.92–6.20)	0.07		
No	6/1868.2	Reference			

*Note:* Poisson regression was used.

Abbreviations: ART, antiretroviral therapy; CI, confidence interval; HBV, hepatitis B; HCV, hepatitis C; IDU, intravenous drug use; IRR, incidence rate ratio; MSM, men who have sex with men; N, number of events; PY, person-year.

**Table 3B.** Univariable and multivariable regression analysis for syphilis incidence

		Univariable		Multivariable	
Characteristic	N/PY	IRR (95% CI)	*p*-value	IRR (95% CI)	*p*-value
IDU	30/268.6	2.20 (1.41–3.44)	<0.001	1.50 (0.94–2.40)	0.09
No	196/3853.0	Reference		Reference	
MSM	213/3696.4	1.94 (0.86–4.35)	0.11	1.18 (0.55–2.54)	0.68
No	13/425.3	Reference		Reference	
Asian	220/3969.7	1.36 (0.48–3.86)	0.56		
No	6/151.9	Reference			
Age (years)	226/4121.6	0.97 (0.95–0.98)	<0.001	0.96 (0.95–0.97)	<0.001
HIV RNA<50 copies/mL at baseline	164/2983.1	1.04 (0.73–1.48)	0.85		
No	62/1138.6	Reference			
CD4 count (100/uL)	226/4121.6	1.00 (0.92–1.08)	0.94		
On ART at baseline	174/3155.3	1.05 (0.72–1.54)	0.80		
No	52/966.3	Reference			
Time from treatment (year)	226/4121.6	0.99 (0.95–1.03)	0.61		
HCV at baseline	12/104.0	2.24 (1.22–4.13)	0.009		
No	214/4017.7	Reference			
HCV new infection (time/year)	226/4121.6	2.16 (0.94–4.94)	0.07		
HBV at baseline	149/2388.2	1.41 (1.00–1.99)	0.053		
No	77/1733.2	Reference			
TPAb at baseline	1772246.4	3.04 (2.00–4.61)	<0.001	3.15 (2.08–4.77)	<0.001
No	49/1875.2	Reference		Reference	

*Note:* Negative binomial regression was used.

Abbreviations: ART, antiretroviral therapy; CI, confidence interval; HBV, hepatitis B; HCV, hepatitis C; IDU, intravenous drug use; IRR, incidence rate ratio; MSM, men who have sex with men; N, number of events; PY, person-year.

For syphilis infection, IDU, younger age, HCV seropositivity at baseline, and TPAb positivity at baseline were associated in univariable analysis. In the multivariable negative binomial regression, younger age (IRR [95% CI], 0.96 [0.95–0.97]/year old, *p* < 0.001) and syphilis seropositivity at baseline (IRR [95% CI], 3.2 [2.1–4.8], *p* < 0.001) were associated with new syphilis infection. We did not perform multivariable analysis for amoebiasis, chlamydia, or gonorrhoea because of the small number of cases.

## Discussion

In the present study, MSM-IDU showed a high seroprevalence of HCV and syphilis at baseline. In addition, the incidence rates of HCV and syphilis were >10 times and two times higher in MSM-IDU than in non-IDU MSM, respectively. In multivariable analysis, MSM, IDU, and new syphilis infections during the study period were associated with a higher HCV incidence. This finding implies that sexual contact may be an important route of HCV transmission in this population.

We found that 6.8% of MSM living with HIV who visited our hospital were injection drug users, which was higher than the 0.5% lifetime experience in the general population in Japan [23]. Nishijima *et al.* reported that approximately 5% of MSM with HIV were injection drug users in the 2000s [21]. While our study was limited to a single centre in Tokyo, it is noteworthy that the proportion of MSM-IDU in our PLWH cohort in the last ten years was not notably distinct from that of the previous report. Further investigation is warranted to assess the longitudinal trends in the proportion of injection drug users among MSM with HIV.

Similar to our study, a high seroprevalence of STIs among MSM-IDU has been reported in other countries [8–11, 25] because MSM-IDU are more likely to engage in high-risk sexual practices, such as condomless sex and group sex, resulting in a higher STI incidence [8]. Crystal methamphetamines have been reported to be associated with STI cases within a year [13]; however, the long-term incidence of STIs among MSM-IDU has not been studied. In the current study, we reported a long-term high incidence of STIs among MSM-IDU. Because reinfection with STIs, especially HCV, is a significant public health concern, estimating the long-term incidence of STIs is essential.

In the present study, the incidence of HCV infection among MSM with HIV was notably high (6.3/1,000 PY), comparable to that of previous HIV cohorts worldwide (5.1–16.3/1,000 PY) [26–28]. This rate is >10 times greater than the global incidence in the general population (0.24/1,000 PY) [29]. Interestingly, the HCV incidence rate among MSM-IDU (44.8/1,000 PY) was >10 times higher than non-IDU MSM (3.5/1,000 PY). Despite the goal set by the World Health Organization (WHO) to reduce the number of new HCV infections by 80% by 2030 (≤ 20/1,000 PY in IDU) [29], the high HCV incidence in our study did not change over time, even in the DAA era (data not shown). Although the treatment success rates were adequately high (94%, 15/16), three cases of reinfection were observed among the MSM-IDU patients. Considering the high cost of DAA, we should strengthen the approach for the HCV high-risk group.

HCV is historically regarded as being more likely to be transmitted by injection drugs, whereas HIV is more likely to be transmitted by male-to-male sexual contact [30, 31]. However, recent data indicate that HCV can also be commonly transmitted through male-to-male sexual contact. In a study based on the administrative claim data, which covered 7 million people in Japan, most HCV or HIV co-infected patients in Tokyo got HIV before HCV [32]. This pattern was commonly seen among MSM, as was observed in this study. Another surveillance study from the Tokyo Metropolitan Infectious Disease Surveillance Centre reported that 33/34 of acute hepatitis C patients in 2019–2021 were male and 31 were MSM [33]. In the present study, MSM, IDU, and new syphilis infections were independently associated with HCV incidence, implying that HCV was transmitted by male-to-male sexual contact and injection drugs among MSM-PLWH. Although it is difficult to distinguish whether HCV is transmitted through injection or sexual contact among MSM-IDU patients, there is no doubt that MSM-IDU is at greater risk of HCV infection among PLWH.

Younger age and a history of syphilis infection were associated with an increased incidence of syphilis. Despite the high incidence of syphilis in the MSM-IDU group, IDU was not a significant independent risk factor for new syphilis infections after adjusting for these two characteristics. As syphilis is unlikely to be transmitted by contaminated needles, this lack of association after adjustment might suggest that MSM-IDU were more likely to be infected with syphilis because they are more sexually active.

The incidence of amoebiasis also tended to be higher in MSM-IDU, but HAV, chlamydia, and gonorrhoea showed no significant differences, partly because of a low number of infections. In a previous study in the United Kingdom, chemsex in the previous year was associated with an increased risk of chlamydia and gonorrhoea infection [34]. The unusually low incidence of chlamydia and gonorrhoea in our study might be attributed to our standard practice that asymptomatic individuals are not tested annually and that Japanese medical insurance covers nucleic acid amplification testing only for urine but not for the anus and pharynx. The seroprevalence of HBV, which is transmitted through both sexual contact and IDU, was higher in the MSM group. It also tended to be higher in MSM-IDUs than in non-IDU MSM; however, the difference was not statistically significant.

Our study had some limitations. Firstly, because methamphetamine is illegal in Japan and the stigma is strong, patients tend to be unwilling to disclose their illicit drug use. Therefore, this study may have underestimated the prevalence of IDU; however, it was still very high. Secondly, HCV Ab tests were not routinely performed at our hospital; thus, the incidence could have been underestimated. However, HCV Ab screening might be of limited value; 575 patients underwent 2,233 HCV Ab tests, and only three new HCV infections were diagnosed by HCV screening. One was seroconverted without liver enzyme elevation, with a very low titre of HCV Ab and negative HCV RNA, which might be a false-positive result for HCV Ab. The remaining 20 new HCV infections were found by elevated liver enzymes, which were tested every 2–3 months in PLWH at our hospital. Although several guidelines have recommended annual HCV Ab testing, even in studies focusing on HCV incidence among PLWH, the practice of annual HCV Ab testing remains uncommon [26–28, 35]. In these studies, liver function tests were performed every 2 to 4 months, and approximately 85–90% of the patients exhibited elevated liver enzymes when HCV Ab became positive [26, 28], which was similar to the present study. Screening tests for syphilis are performed regularly, even in asymptomatic patients. Thirdly, as this study included only PLWH, the results may not be applicable to non-HIV MSM.

We found a high prevalence of IDU among MSM with HIV and a high incidence of HCV and syphilis among MSM-IDU with HIV in Tokyo, Japan. The HCV incidence in MSM-IDU was 10 times higher than that in non-IDU MSM and was associated with MSM, IDU, and new syphilis infection. There is a need to appropriately identify MSM-IDU and take action to prevent STI outbreaks in this high-risk group.

## Data Availability

The data are available upon request. The data were collected from routine clinical records. The study data set with de-identified participant data is available at yotsudid@ims.u-tokyo.ac.jp.
